# Cell Painting Identifies Phenotypic Clusters Associated with Anticancer Drug Responses in OVCAR-3 Ovarian Cancer Cells

**DOI:** 10.3390/ijms27198791

**Published:** 2026-09-30

**Authors:** Vladimir E. Popov, Velemir S. Lavrinenko, Ekaterina V. Litau, Alena A. Shurova, Mikhail A. Trofimov, Daria M. Danilenko, Dmitry A. Lioznov, Ramziya G. Kiyamova, Stanislav F. Tyazhelnikov

**Affiliations:** 1 JSC BIOCAD, 191186 Saint Petersburg, Russia; lavrinenkovelemir@gmail.com (V.S.L.); kkaterina.abc@gmail.com (E.V.L.); shurovaalyona2003@gmail.com (A.A.S.); 2 Biomarker Research Laboratory, Institute of Fundamental Medicine and Biology, Kazan Federal University, 420008 Kazan, Russia; kiyamova@mail.ru; 3 Nanotechnology Research and Education Centre, Saint Petersburg Academic University, 194021 Saint Petersburg, Russia; mih.andr.trofimov@gmail.com; 4 Smorodintsev Research Institute of Influenza, 197376 Saint Petersburg, Russia; daria.baibus@gmail.com (D.M.D.); dlioznov@yandex.ru (D.A.L.); 5 Department of Infectious Diseases and Epidemiology, Pavlov First Saint Petersburg State Medical University, 197022 Saint Petersburg, Russia

**Keywords:** cell painting, morphological profiling, ovarian cancer, OVCAR-3, phenotypic screening, mechanism of action, unsupervised clustering

## Abstract

Cell Painting is a high-content imaging approach that enables systematic characterization of cellular responses to chemical perturbations through quantitative morphological profiling. Although this methodology has been widely applied in drug discovery and functional genomics, ovarian cancer models remain comparatively underrepresented in Cell Painting studies. In the present work, we established a Cell Painting workflow for OVCAR-3 ovarian cancer cells and applied it to the phenotypic profiling of 54 anticancer compounds representing diverse mechanisms of action. Cells were exposed to compounds under short- and prolonged-treatment conditions, followed by multiplex fluorescent staining, high-content imaging, feature extraction using CellProfiler, and computational analysis of the resulting morphological profiles. Profiles obtained at the two treatment durations were analyzed separately. For each compound, the treatment condition showing greater within-compound consistency in PCA space was additionally retained to construct a combined dataset. Morphological relationships were explored using Pearson correlation analysis, dimensionality reduction, and unsupervised clustering. Morphological profiles showed reproducible relationships among compounds. Compounds with related mechanisms showed greater morphological similarity than expected by chance, although substantial within-class heterogeneity remained. Statistically significant cluster enrichment was observed for microtubule-targeting agents and topoisomerase inhibitors, whereas other mechanism-related patterns were incomplete. Among the evaluated clustering approaches, Leiden and agglomerative clustering produced similar separation of the Combined dataset, with silhouette scores of 0.486 and 0.482, respectively. Cell Painting identified mechanism-associated but heterogeneous morphological responses in OVCAR-3 cells. The results support the use of this model for exploratory phenotypic profiling while highlighting the influence of exposure conditions and the limited ability of morphology alone to recover annotated molecular mechanisms.

## 1. Introduction

Despite major advances in molecularly targeted therapies, predicting cellular responses to anticancer compounds remains challenging because drug activity emerges from complex interactions among signaling pathways, metabolic processes, and stress-response mechanisms rather than from modulation of a single molecular target. This challenge is particularly relevant in cancer, where genetic heterogeneity, adaptive signaling, and context-dependent responses frequently influence therapeutic outcomes. Phenotypic profiling has re-emerged as an important strategy in drug discovery because it directly captures integrated cellular responses to perturbations instead of relying exclusively on predefined molecular targets. By measuring system-level consequences of chemical or genetic perturbations, phenotypic approaches can reveal both expected and unexpected mechanisms of action, identify phenotypic convergence between distinct compounds, and provide insights into broader cellular response patterns associated with therapeutic response [1,2,3,4,5,6,7].

Among image-based phenotypic profiling approaches, Cell Painting has become one of the most widely adopted methods for high-content characterization of cellular states. The assay combines multiplex fluorescent staining of multiple cellular compartments with automated microscopy and large-scale feature extraction, generating rich quantitative representations of cellular morphology [1]. Since its introduction, Cell Painting has evolved into a broadly used platform for perturbation biology, supported by continuous advances in image analysis, machine learning, and large public reference resources such as the JUMP Cell Painting consortium [2,4,8,9,10]. The resulting morphological profiles have been successfully applied to compound annotation, mechanism-of-action studies, toxicity assessment, functional genomics, variant interpretation, and prediction of biological activity [2,3,4,5,9,10,11,12,13,14].

A key advantage of Cell Painting is its ability to capture integrated cellular phenotypes that extend beyond canonical molecular targets. Compounds acting on distinct proteins or pathways may nevertheless induce similar morphological signatures if they converge on related biological processes such as replication stress, DNA damage responses, mitotic arrest, metabolic adaptation, or suppression of proliferative signaling. Conversely, compounds targeting the same pathway may generate distinct phenotypes depending on cellular context and the downstream consequences of pathway inhibition. As a result, morphology-based profiling can reveal both target-proximal and target-distal relationships among perturbations, providing a complementary perspective to traditional target-centric approaches [2,4,5,11,12,15].

These properties are particularly relevant in oncology. Ovarian cancer remains the most lethal gynecologic malignancy, largely due to late-stage diagnosis, extensive molecular heterogeneity, and the frequent development of resistance to chemotherapy and targeted therapies. Although substantial progress has been achieved through the introduction of PARP inhibitors and other targeted agents, treatment responses remain highly variable and are influenced by multiple interconnected biological processes. Such complexity makes ovarian cancer an attractive setting for phenotypic profiling approaches capable of capturing integrated cellular responses to pharmacological perturbations. At the same time, ovarian cancer models remain comparatively underrepresented in Cell Painting studies and in large-scale morphology-based reference datasets, limiting our understanding of how anticancer compounds are organized in phenotypic space in this disease context [5,16]. In particular, relatively little is known about whether morphology-based profiling in ovarian cancer cells can resolve mechanism-related phenotypic states and how strongly such states depend on treatment duration and analytical strategy.

OVCAR-3 is a widely used ovarian adenocarcinoma cell line that retains multiple characteristics relevant to epithelial ovarian cancer biology and therapeutic response. At the same time, its growth properties and morphological characteristics distinguish it from the cellular models most commonly used in large-scale Cell Painting studies. Establishing morphology-based profiling workflows in additional cellular contexts is therefore important for expanding the biological scope of Cell Painting. Recent studies have further emphasized that cellular context, feature extraction strategy, and analytical methodology can substantially influence the interpretation of image-based phenotypes in ovarian cancer systems [16]. These considerations make OVCAR-3 a relevant model both for technical adaptation of the assay and for examining how anticancer perturbations are represented in the morphological feature space of ovarian cancer cells.

Reliable Cell Painting studies also require careful experimental design. Factors such as cell density, treatment duration, compound concentration, staining conditions, and image acquisition parameters directly affect both image quality and biological interpretability of the resulting profiles [2,4,5]. In addition, drug-induced morphological responses are inherently dynamic. The same compound may produce distinct phenotypes at different stages of exposure, reflecting direct target engagement, downstream adaptation, cumulative stress, or progressive loss of viability. Incorporating temporal information into phenotypic profiling workflows may provide a more comprehensive representation of compound activity than analysis at a single endpoint [1,5,15]. This issue is especially relevant when comparing compounds with distinct response kinetics, because short-term and prolonged exposure may capture different layers of the cellular response.

The high-dimensional nature of Cell Painting data further necessitates computational approaches capable of identifying major sources of phenotypic variation and organizing compounds according to profile similarity. Correlation analysis, dimensionality reduction, and unsupervised clustering are therefore widely used to explore relationships among perturbations without imposing predefined biological categories [5,10,15,17]. In particular, graph-based clustering approaches such as Leiden have attracted increasing attention because they can capture local neighborhood structure and complex phenotypic relationships that may not be adequately represented by conventional centroid-based methods [5,17]. However, the relative utility of different clustering strategies may depend on the biological system, profile structure, and preprocessing choices, making system-specific evaluation important.

In the present study, we established a Cell Painting workflow for the ovarian cancer cell line OVCAR-3 and applied it to phenotypic profiling of 54 anticancer compounds representing diverse mechanisms of action. We aimed not only to implement the assay in this disease-relevant model, but also to characterize how drug-induced morphological responses are organized in OVCAR-3 cells, to assess the contribution of treatment duration to phenotypic coherence, and to determine whether unsupervised analysis can resolve biologically interpretable phenotypic states associated with distinct classes of anticancer compounds. To this end, we analyzed morphological profiles generated after short and prolonged drug exposure, integrated temporal information into a unified phenotypic dataset, and evaluated several unsupervised clustering strategies for identifying robust phenotypic states.

## 2. Results

### 2.1. Establishment of Cell Painting Profiling Conditions in OVCAR-3 Cells

Cell Painting was applied to OVCAR-3 ovarian cancer cells to characterize morphological responses to a panel of anticancer compounds with diverse mechanisms of action. Multiparametric image-derived profiles were generated across the full compound panel, providing a high-dimensional representation of treatment-associated cellular states. Because the assay simultaneously captured features from multiple cellular compartments and structures, the resulting profiles reflected integrated phenotypic responses rather than isolated single-parameter effects.

Across the eight primary Cell Painting datasets, 19,008 image sets were acquired. Of these, 396 image sets (2.08%) were excluded because of insufficient image sharpness and 127 (0.67%) because fewer than 20 nuclei were detected, resulting in a total exclusion rate of 2.75%. DNA-channel saturation was monitored separately and was detected in 29 image sets (0.15%) but was not used as an independent exclusion criterion.

Image loss was not uniformly distributed across compounds. DMSO controls showed an exclusion rate of 1.74%, whereas the highest compound-specific rates were observed for vincristine (11.81%), tegafur (10.07%), crizotinib (7.99%), vandetanib (7.29%), and osimertinib (6.60%). Thus, although overall QC-related image loss was low, selective loss of fields in individual treatment conditions cannot be completely excluded as a potential source of bias.

The compound panel generated a broad range of morphological responses, including profiles that reproducibly departed from the DMSO control distribution and profiles for which no reproducible response distinguishable from DMSO was detected under the tested conditions.

### 2.2. Drug-Induced Morphological Responses Generate a Structured Phenotypic Space

**Figure 1:**
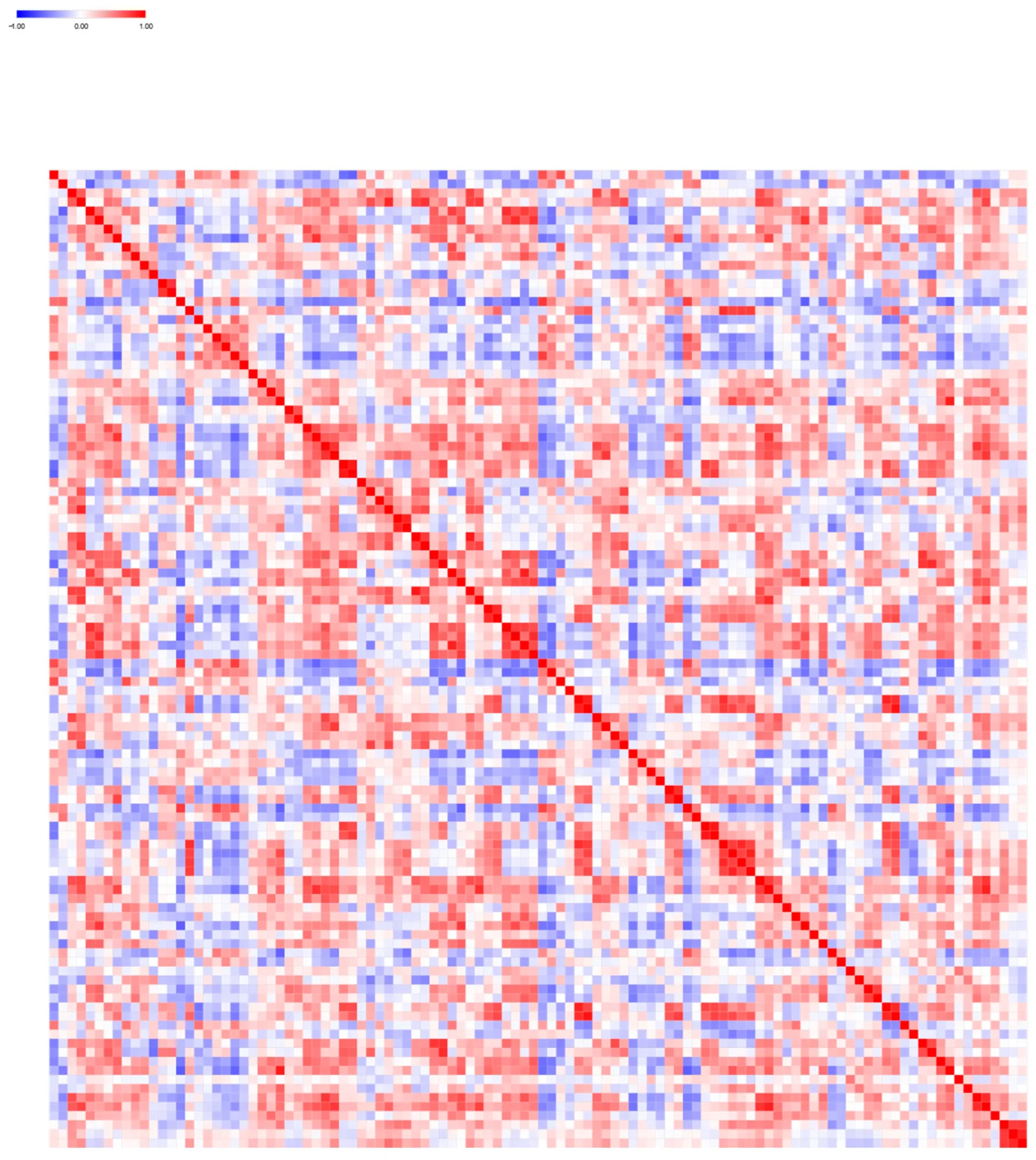
Pearson correlation heatmap of Cell Painting-derived morphological profiles after short-term compound treatment. Axis labels were omitted for readability because of the large number of profiles. Profiles are displayed in the same order on both axes. The corresponding fully labeled vector heatmap is available in the public Zenodo repository (https://doi.org/10.5281/zenodo.22958654). Morphological similarity is interpreted here as phenotypic convergence and does not by itself demonstrate engagement of the compounds’ annotated primary targets.

After illumination correction and quality control, CellProfiler generated high-dimensional morphological profiles for the analyzed compound panel. Pearson correlation analysis revealed a structured pattern of relationships among compound-associated morphological profiles after short-term exposure (Figure 1). A comparable correlation pattern was also observed after prolonged incubation (Supplementary Figure S3), supporting the use of dimensionality reduction prior to clustering. Such correlation structure is expected in Cell Painting data, where partially overlapping descriptors of shape, intensity, and texture often capture related aspects of cellular state [5,12,15].

Several of the observed phenotypic similarities were consistent with known mechanisms of action. For example, niraparib and olaparib showed convergent morphological profiles, in agreement with their shared effects on DNA repair and replication stress [18,19]. Similarity was also observed between microtubule-targeting agents, particularly vinblastine and vincristine, which is consistent with their common effects on cytoskeletal organization and mitotic progression.

Additional relationships were identified among compounds targeting receptor tyrosine kinase signaling. Afatinib showed morphological similarity to entrectinib, erlotinib, gefitinib, and osimertinib, consistent with the fact that these compounds suppress proliferative and survival-associated signaling pathways. Erlotinib, gefitinib, afatinib, and osimertinib inhibit EGFR, whereas entrectinib targets TRK, ROS1, and ALK, which are likewise involved in the maintenance of mitogenic and anti-apoptotic signaling [20,21,22]. Partial phenotypic similarity was also observed among inhibitors of the EGFR/HER family, the PI3K/mTOR axis, and other receptor tyrosine kinases, suggesting convergence of these perturbations on related antiproliferative cellular states.

**Figure 2:**
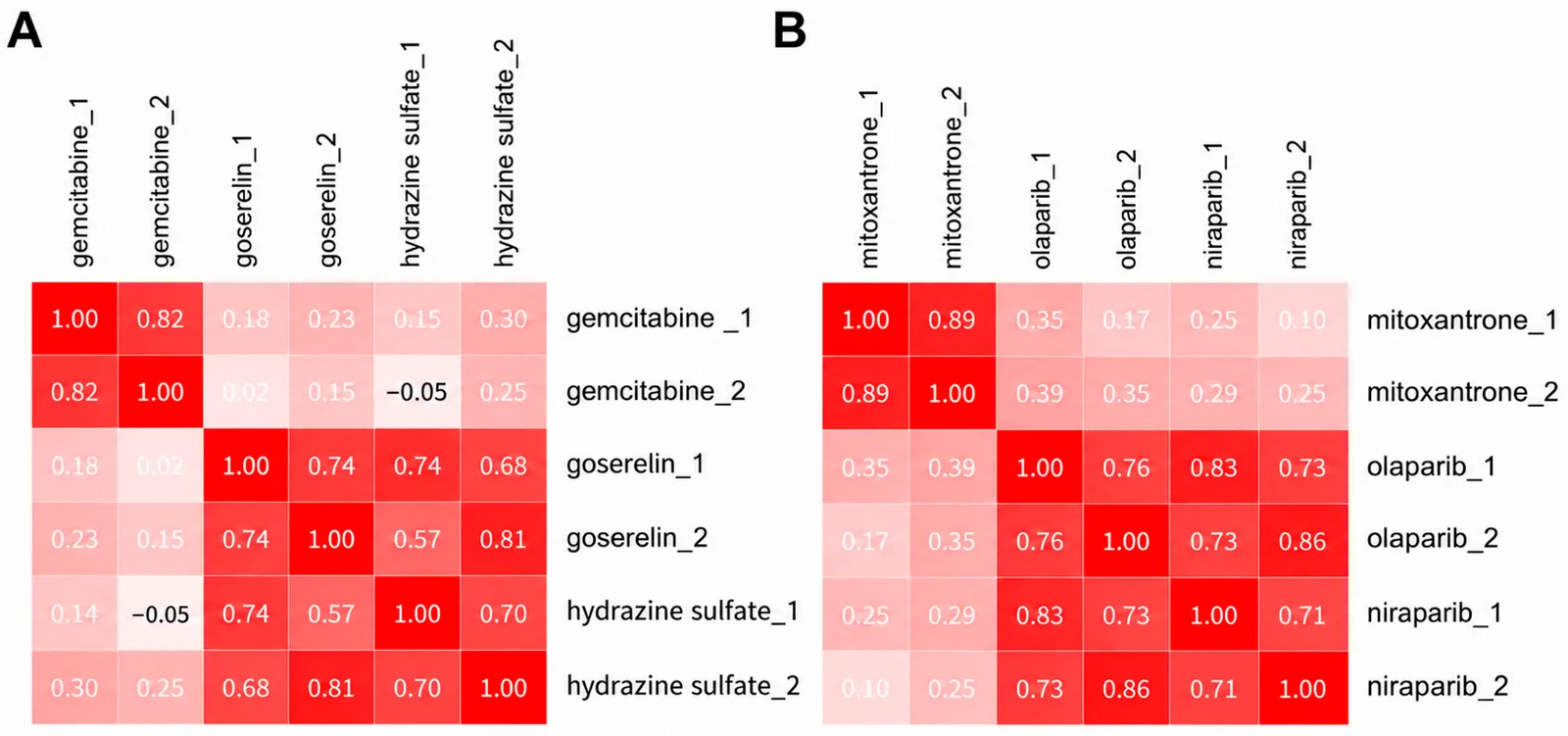
Fragment of the heatmap of Pearson correlation coefficients for morphological features obtained by the Cell Painting assay after short-term compound exposure. The heatmap shows the exact values of the Pearson correlation coefficients between the morphological profiles of the studied compounds. (**A**) Pairwise correlation analysis of morphological profiles induced by gemcitabine, goserelin, and hydrazine sulfate. (**B**) Pairwise correlation analysis of morphological profiles induced by niraparib, olaparib, and mitoxantrone. Color intensity represents the Pearson correlation coefficient, with darker red indicating stronger positive correlation and lighter shades indicating weak or near-zero correlation. Morphological similarity is interpreted here as phenotypic convergence and does not by itself demonstrate engagement of the compounds’ annotated primary targets.

Not all prominent correlations were limited to compounds with closely related annotated targets. High morphological similarity was also observed between goserelin and hydrazine sulfate despite their different mechanism annotations (Figure 2). Such observations indicate that Cell Painting captures not only target-proximal effects, but also broader cellular response patterns shared by mechanistically distinct compounds.

These results showed that several compounds with related annotated mechanisms produced similar morphological profiles, while additional similarities were observed between compounds acting on different molecular targets. Accordingly, these phenotypic similarities should be interpreted as morphological convergence rather than as direct evidence of engagement of the annotated primary targets.

### 2.3. Phenotypic Profiles Differ Between the Two Exposure Conditions

Drug-induced morphological responses differed between short and prolonged exposure. Phenotypic profiles were therefore analyzed separately for the two treatment-duration conditions and were additionally integrated into a combined dataset. For each compound, the treatment condition showing the lower mean pairwise distance among replicate-well profiles in PCA space was retained as the more internally consistent representation.

The 2-day and 8-day experimental conditions differed not only in exposure duration but also in initial seeding density (3000 versus 500 cells per well, respectively). These factors are therefore confounded in the present design, and differences between the two conditions cannot be attributed exclusively to treatment duration.

The combined dataset was used as an integrative representation of the two treatment durations for subsequent comparison of clustering approaches. Because treatment duration was selected using within-compound profile consistency, the combined dataset was not used as evidence that this selection procedure outperformed either single-duration dataset. Differences between the 2- and 8-day profiles nevertheless indicated that the two exposure conditions produced distinct morphological responses in OVCAR-3 cells.

DMSO-based activity assessment showed that, at the exposure duration selected for the Combined dataset, 35 of 54 compounds produced a reproducible morphological response in both biological replicates, 7 were active in one replicate, and 12 showed no reproducible morphological response distinguishable from DMSO in either replicate.

Importantly, only one compound, tegafur, was assigned to an exposure duration without a detectable response despite showing reproducible morphological activity at the alternative duration. Eleven of the 12 compounds lacking a reproducible response at the selected duration were also non-responsive at the alternative duration. The reproducibility-based duration-selection procedure did not systematically favor inactive profiles.

In the DMSO-inclusive diagnostic analysis, all eight dataset-level DMSO profiles grouped together, while the drug-only cluster assignments remained highly stable (adjusted Rand index = 0.972 relative to the primary solution) (Supplementary Figure S4).

To determine whether inclusion of non-responsive compounds materially influenced the clustering solution, the 12 compounds showing no reproducible response at the selected exposure duration were excluded and the fixed Leiden analysis was repeated on the remaining 42 compounds. The resulting solution retained five clusters, yielded a silhouette score of 0.508, and showed one split compound. Cluster assignments for the retained compounds were unchanged up to label permutation (adjusted Rand index = 1.0).

The selected exposure duration contributed to the organization of the Combined phenotypic space. Olaparib was represented by its 8-day profile, whereas niraparib and mitoxantrone were represented by their 2-day profiles. The separation of olaparib from niraparib in the Combined clustering may partly reflect exposure-condition differences, whereas the co-clustering of niraparib and mitoxantrone cannot be attributed to exposure duration alone.

### 2.4. Unsupervised Analysis Reveals Major Phenotypic States in OVCAR-3 Cells

Leiden, k-means, and agglomerative clustering were compared on the Combined dataset using the grid-search procedure described in Methods.

**Figure 3:**
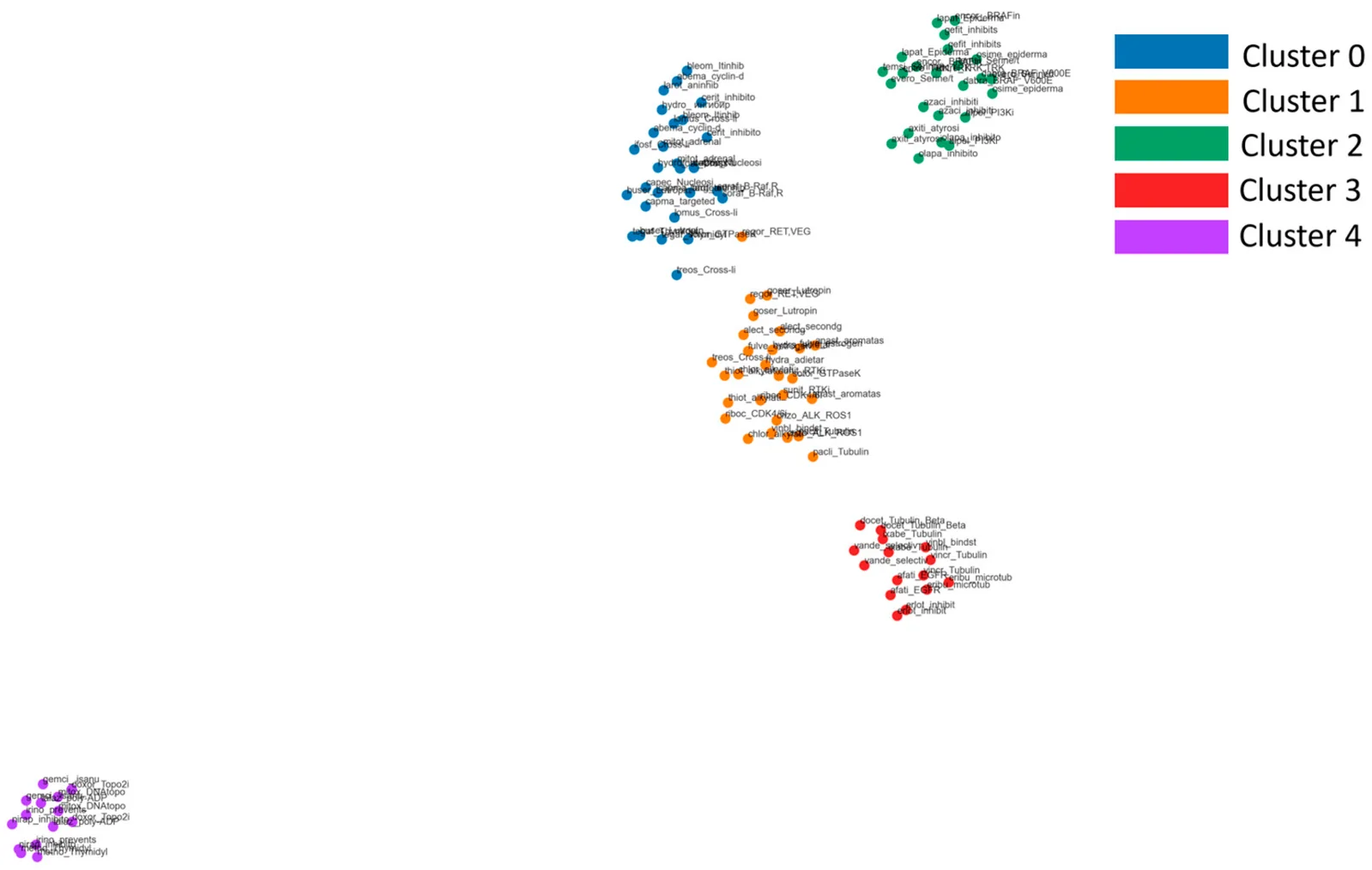
UMAP visualization of the combined OVCAR-3 Cell Painting dataset clustered with the Leiden algorithm. The combined dataset was generated by retaining, for each compound, the treatment-duration condition with the lower mean pairwise distance among replicate-well profiles in PCA space. The Leiden clustering solution shown here was obtained using cosine distance, 50 principal components, 10 nearest neighbors, and a resolution value of 0.40, yielding 5 clusters with a silhouette score of 0.486 and 3 split compounds.

Among the evaluated approaches, Leiden clustering yielded the highest silhouette score for the combined dataset. The best-performing configuration used cosine distance, 50 principal components, 10 nearest neighbors, and a resolution of 0.40, yielding 5 clusters with a silhouette score of 0.486 and 3 split compounds (Figure 3). In these split compounds, the two biological-replicate profiles were assigned to different clusters. Compound-level Leiden cluster assignments are provided in Supplementary Table S1.

Leiden and agglomerative clustering produced quantitatively similar separation of the Combined dataset, with full-data silhouette scores of 0.486 and 0.482, respectively, whereas the selected k-means solution contained six clusters and yielded a silhouette score of 0.408. Across 1000 repeated 80% compound-level subsamples, mean silhouette scores were 0.487 ± 0.015 for Leiden, 0.483 ± 0.016 for agglomerative clustering, and 0.408 ± 0.025 for k-means. The mean Leiden–agglomerative difference was only 0.0039, substantially smaller than the dispersion observed across subsamples.

Inspection of cluster composition showed that some clusters contained compounds acting on related biological processes, whereas others were mechanistically heterogeneous. Cluster 2 contained several targeted signaling inhibitors. It included osimertinib, a third-generation EGFR inhibitor active against sensitizing and T790M resistance mutations [20]; gefitinib, a classical EGFR tyrosine kinase inhibitor [23]; dabrafenib and encorafenib, which inhibit mutant BRAF and suppress MAPK signaling [24,25]; alpelisib, a PI3Kα-selective inhibitor [26]; everolimus and temsirolimus, which target mTOR signaling [27,28]; and axitinib, an inhibitor of VEGFR-associated signaling [29]. Additional members of this cluster included entrectinib, lapatinib, olaparib, and azacitidine.

The overall composition of Cluster 2 suggests convergence on a shared morphological state associated with suppression of proliferative and survival-related cellular programs. The presence of olaparib and azacitidine in this cluster indicates that it is not specific to one signaling pathway. The shared morphology may instead reflect downstream effects of growth inhibition or cellular stress.

Cluster 3 was dominated by compounds that disrupt microtubule dynamics and mitotic progression, together with a smaller subset of inhibitors targeting the EGFR family. It included docetaxel, a taxane that stabilizes microtubules [30]; ixabepilone, an epothilone analog [31]; eribulin, an inhibitor of microtubule growth [32]; and vincristine, a vinca alkaloid that promotes microtubule destabilization [33,34]. The emergence of a distinct microtubule-associated cluster highlights the sensitivity of morphology-based profiling to perturbations affecting cytoskeletal organization and mitotic progression.

In addition to direct tubulin-targeting agents, this cluster also contained afatinib [22], vandetanib [35], and erlotinib [36]. The co-occurrence of microtubule-directed compounds and selected EGFR/HER family inhibitors may reflect partial phenotypic convergence rather than shared primary target identity. Changes in cell shape, adhesion, cytoskeletal organization, and cell-cycle progression may represent common downstream consequences of both direct perturbation of the microtubule apparatus and suppression of proliferative signaling pathways.

Cluster 4 contained several compounds associated with DNA damage and replication stress and showed statistically significant enrichment for topoisomerase inhibitors. This group included doxorubicin [37], mitoxantrone [38], irinotecan [39], gemcitabine [40], methotrexate [41], and the PARP inhibitors niraparib and talazoparib [18,19,42]. It represented one of the most biologically coherent groups in the dataset, bringing together agents that directly or indirectly impair genome maintenance and increase genotoxic or replicative stress. The grouping of these compounds is consistent with their known effects on genome integrity and suggests that DNA damage and replication stress produce related morphological responses in OVCAR-3 cells.

The remaining clusters were more heterogeneous in composition than the more clearly defined signaling-, microtubule-, and DNA damage-associated groups. Cluster 0 included classical alkylating and DNA-reactive agents such as ifosfamide and lomustine [43,44,45,46,47], as well as bleomycin [48], hydroxycarbamide [49], capecitabine and tegafur [50,51]; kinase inhibitors including capmatinib [52], larotrectinib [53], ceritinib [54], abemaciclib [55], and sorafenib [56]; the hormonal therapy agent buserelin [57]; and the adrenolytic agent mitotane [58]. The heterogeneous composition of Cluster 0 did not support assignment to a single mechanism of action. Its shared morphology may instead reflect common downstream effects on proliferation, cellular stress, or other treatment-associated processes.

Another heterogeneous group, Cluster 1, included alkylating agents such as chlorambucil, thiotepa, and treosulfan [43,44,45,46,47]; endocrine therapies including anastrozole [59], fulvestrant [60], and goserelin [57]; the CDK4/6 inhibitor ribociclib [61]; and the multikinase inhibitors regorafenib [62], alectinib [63], crizotinib [64], and sunitinib [65]. This cluster also contained hydrazine sulfate, as well as paclitaxel, a microtubule-stabilizing taxane [66], further emphasizing its mixed composition. The heterogeneous composition of Cluster 1 likewise did not support assignment to a single target class. The observed morphological similarity may reflect common effects on proliferation, cellular stress, or cellular organization.

Formal enrichment testing identified two cluster–mechanism associations that remained significant after FDR correction: microtubule-targeting compounds in Cluster 3 (4/7 compounds; FDR-adjusted *p* = 0.030) and topoisomerase inhibitors in Cluster 4 (3/7 compounds; FDR-adjusted *p* = 0.038). Other visually apparent mechanism associations, including signaling inhibitors in Cluster 2, did not remain significant after correction and are therefore described descriptively rather than as statistically significant enrichment. Complete results of the cluster–mechanism enrichment analysis, including all Fisher exact tests and FDR-adjusted *p* values, are provided in Supplementary Table S2.

Replicate reproducibility was substantially greater than expected by chance: 34 of 54 compounds (63.0%) met the replicate-similarity criterion, compared with a mean of 5.1% in the empirical null distribution (95th percentile, 9.3%; empirical *p* = 0.0002). Same-mechanism retrieval was also greater than expected by chance, with an observed mean average precision (mAP) of 0.378 compared with a shuffled-label mean of 0.226 (95th percentile, 0.260; permutation *p* = 0.0002). Together, these results indicate that the morphological profiles were reproducible and contained statistically detectable, although incomplete, information related to annotated pharmacological mechanisms.

To assess whether the observed clustering primarily reflected experimental structure, the Combined UMAP was recolored according to compound cohort, plate, biological replicate, and selected exposure duration (Figure S5). Biological replicates were evenly distributed across the phenotypic clusters, and no cluster corresponded uniquely to a single compound cohort or individual plate. In contrast, a marked association with selected exposure duration was observed: Cluster 2 consisted exclusively of 8-day profiles, whereas Clusters 3 and 4 consisted exclusively of 2-day profiles. Because plates were duration-specific in the experimental design, plate identity and exposure duration cannot be fully disentangled. The clustering was not explained by biological replicate or a single cohort effect, but exposure-condition structure contributed substantially to the organization of the Combined phenotypic space.

Although several mechanism-related patterns were apparent, compounds with similar annotated mechanisms did not invariably occupy the same Leiden cluster. For example, docetaxel and paclitaxel were assigned to different clusters, vinblastine showed split replicate assignments despite the clustering of other microtubule-targeting agents, and olaparib was separated from niraparib and talazoparib. Discordant assignments were also observed among CDK4/6 and several kinase inhibitors. These discordances are consistent with the quantitative retrieval analysis, indicating that the relationship between morphological phenotype and annotated mechanism of action was statistically detectable but incomplete.

Inclusion of the third 8-day biological replicate produced a Combined dataset containing 131 biological-replicate profiles and retained a five-cluster solution with a silhouette score of 0.487 and four split compounds. Among 106 profiles shared with the primary analysis, cluster assignments remained highly similar (adjusted Rand index = 0.972). Only one compound, mitotane, changed its selected exposure duration from 2 days to 8 days. When the two primary biological-replicate profiles were further aggregated to a single median profile per compound, the compound-level silhouette score was 0.545, indicating that the observed cluster separation was not dependent solely on the paired-replicate structure of the 108-profile dataset. The results of these sensitivity analyses are summarized in Supplementary Table S3.

### 2.5. Agglomerative Clustering

Agglomerative clustering was evaluated across 75 parameter combinations. The best-performing solution used cosine distance, 50 principal components, and 5 clusters, yielding a silhouette score of 0.482 and 4 split compounds. This performance was quantitatively close to that of the optimal Leiden solution (Figure S6).

Agglomerative clustering recovered the same major biological patterns identified by Leiden clustering, including signaling-associated, microtubule-associated, and DNA damage-associated phenotypic groups. This agreement indicates that these patterns were not specific to the Leiden algorithm.

### 2.6. K-Means Clustering

The best-performing k-means configuration used 50 principal components and 6 clusters. For this configuration, the corresponding combined dataset was constructed using cosine distance and yielded a cosine-based silhouette score of 0.408 with 6 split compounds (Figure S7).

K-means clustering recovered several of the major biological themes observed with Leiden and agglomerative clustering, including groups containing signaling inhibitors, DNA damage-associated compounds, and microtubule modulators. However, cluster composition was generally more heterogeneous and quantitative performance was lower.

Across the three clustering approaches, signaling-, microtubule-, and DNA damage-associated groups were recovered most consistently, whereas the remaining clusters were more mechanistically heterogeneous. Leiden produced the highest silhouette score among the evaluated methods, although agglomerative clustering yielded a quantitatively similar solution.

## 3. Discussion

Cell Painting profiling of 54 anticancer compounds in OVCAR-3 cells identified reproducible morphological relationships, including groups containing signaling inhibitors, microtubule-targeting agents, and compounds associated with DNA damage or replication stress. Other clusters were mechanistically mixed, showing that morphological similarity did not always follow annotated molecular targets.

Ovarian cancer is characterized by marked molecular heterogeneity and variable responses to systemic therapies, making it a particularly relevant context for phenotypic profiling approaches. Although Cell Painting has been widely applied across different biological systems, ovarian cancer-specific morphological datasets remain comparatively limited. In this context, the present work extends morphology-based profiling to the OVCAR-3 ovarian cancer cell model and provides a dataset for examining drug-induced morphology in this defined cellular context.

The two exposure conditions produced distinct morphological profiles. To integrate these conditions, the combined dataset retained, for each compound, the treatment duration showing greater within-compound profile consistency. This approach provided a practical representation for exploratory clustering of a pharmacologically diverse compound panel. However, because treatment duration was selected using the same profile-consistency criterion used to construct the dataset, the combined dataset should not be interpreted as an independent demonstration of improved clustering performance relative to either single-duration condition.

The assay medium also differed from the routine propagation medium by containing lower serum and no supplemental insulin. This condition was applied uniformly to compound-treated and DMSO-control wells and therefore defines the assay-specific baseline used for all DMSO-normalized comparisons. Prolonged serum and insulin restriction may itself alter the baseline OVCAR-3 phenotype, particularly during the 8-day condition.

No medium was replaced during the 8-day exposure; therefore, nutrient depletion and other time-dependent changes in the culture environment may also have contributed to the prolonged-condition phenotype. Because nuclear count was not modeled as an independent covariate, treatment-associated differences in cell density may have contributed to some morphological profiles, particularly for strongly cytotoxic compounds.

Although only 2.75% of image sets were excluded overall, QC losses were higher for several individual compounds than for DMSO controls. Selective survival of analyzable fields, particularly under strongly perturbing or cytotoxic conditions, remains a potential source of bias. The complete per-compound distribution of QC-related image exclusions is reported in Supplementary Table S4.

The combined dataset should therefore be interpreted as an exploratory representation integrating the most reproducible phenotype observed for each compound rather than as a comparison performed under a single uniform experimental condition. Because exposure duration was selected independently for each compound and was coupled to different initial seeding densities, both treatment duration and experimental context may contribute to cluster separation.

Comparison with DMSO-based activity calls indicated that the selection procedure did not systematically favor inactive profiles: only one of 54 compounds was assigned to a non-responsive duration despite showing reproducible activity at the alternative duration.

Not all compounds produced reproducible morphological responses distinguishable from DMSO under the tested conditions. These compounds were retained in the primary clustering analysis but were explicitly flagged in the Supplementary Table S1. A sensitivity analysis excluding the 12 non-responsive selected profiles reproduced the cluster organization of the retained compounds, indicating that the primary clustering solution was not driven solely by compounds close to the vehicle baseline. Cluster membership of compounds without a reproducible activity call should not be interpreted as evidence of a mechanism-specific drug response.

Several clusters showed partial correspondence with annotated mechanisms of action, but this relationship was neither uniform nor deterministic. The DNA damage-associated cluster represented one of the most coherent groups in the dataset and included PARP inhibitors together with other compounds linked to genome maintenance pathways. This observation is consistent with previous reports showing that genotoxic stress produces characteristic and reproducible morphological signatures detectable by Cell Painting. Likewise, compounds affecting microtubule dynamics formed a distinct phenotypic group despite differences in their direct interactions with the cytoskeleton, highlighting the sensitivity of morphology-based profiling to perturbations affecting cellular architecture and mitotic progression. A separate cluster contained several inhibitors of EGFR, BRAF, PI3K, mTOR, and VEGFR signaling, although this association did not remain significant after FDR correction.

Not all clusters were associated with a single mechanism of action. Several included compounds acting on different pathways, suggesting that morphological similarity does not necessarily reflect shared molecular targets. Instead, these compounds may produce similar downstream effects on proliferation, cellular stress, or cellular organization.

Leiden and agglomerative clustering produced quantitatively similar solutions. Although Leiden had the numerically highest full-data silhouette score (0.486 versus 0.482), the difference was small relative to the variability observed across repeated compound-level subsampling and should not be interpreted as demonstrating superiority of Leiden over agglomerative clustering.

The observed discordances indicate that morphology-based similarity in OVCAR-3 cells does not provide a one-to-one readout of annotated molecular mechanisms. Compounds sharing a nominal target class may generate distinct downstream cellular states because of differences in target engagement, pathway context, exposure duration, concentration, or technical variability. Conversely, mechanistically distinct compounds may converge on similar morphological states. The significant but moderate same-mechanism retrieval performance observed here therefore supports partial mechanism-related organization rather than deterministic recovery of the mechanism of action.

An important limitation of the compound panel is that not all agents would be expected to exert their canonical pharmacological activity in OVCAR-3 cells under the conditions used here. Several compounds require metabolic activation or depend on specific molecular or hormonal contexts for their canonical effects. For example, capecitabine is a prodrug that requires conversion to 5-fluorouracil [50], whereas the canonical activities of capmatinib and ceritinib are associated with MET-altered and ALK-positive tumor contexts, respectively [52,54]. Similarly, the activity of endocrine agents such as anastrozole and GnRH-directed therapies depends on the corresponding hormone-responsive signaling context [57,59]. Consistent with these considerations, capecitabine, ifosfamide, sotorasib, capmatinib, ceritinib, anastrozole, buserelin, and treosulfan showed no reproducible morphological response distinguishable from DMSO under either exposure condition. These compounds should therefore not be interpreted as defining mechanism-specific phenotypic states.

Conversely, the presence of a reproducible morphological response does not necessarily demonstrate engagement of the compound’s canonical therapeutic mechanism. For example, several agents with context-dependent or activation-dependent pharmacology produced detectable morphological responses under one or both exposure conditions. Such responses may reflect cell-autonomous metabolism, off-target activity, pathway-context effects, or other downstream consequences of compound exposure. Accordingly, the pharmacological annotations used in this study should be regarded as reference mechanism labels rather than evidence that the corresponding canonical targets were engaged in OVCAR-3 cells.

Because selected exposure duration was strongly associated with parts of the Combined phenotypic space, and because plate identity was nested within exposure duration, the resulting clusters cannot be interpreted as purely mechanism-driven structures.

Several limitations should be considered when interpreting these findings. First, the study was performed in a single ovarian cancer cell line using a moderate-sized compound panel, limiting generalization to other cellular models or treatment contexts. Second, morphological similarity does not necessarily imply an identical mechanism of action, and heterogeneous clusters may reflect common response intensity, cytostatic effects, or other non-specific consequences of treatment. Because compound concentrations were individually selected and, for some compounds, differed between the 2-day and 8-day exposure conditions, concentration represents an additional source of heterogeneity in the dataset. Consequently, differences in morphological profile magnitude or cluster assignment cannot be attributed exclusively to molecular mechanisms. Third, the study did not include orthogonal molecular or functional validation of the inferred phenotypic relationships. Future studies could further extend Cell Painting phenotypes by integrating morphological profiles with complementary cellular measurements, including cell number and viability, pathway-specific molecular markers, and transcriptomic or proteomic readouts. Such multimodal analyses may help distinguish morphological convergence caused by shared molecular mechanisms from more general effects associated with proliferation, cytotoxicity, or cellular stress. Fourth, the combined dataset was constructed by selecting treatment duration according to within-compound profile consistency and should therefore be considered an exploratory integrative representation rather than an independent validation of the selection strategy. Additional cellular models, complementary assays, and denser temporal sampling will be useful for testing the generality of these observations.

## 4. Materials and Methods

### 4.1. Cell Line and Culture Conditions

The human ovarian adenocarcinoma cell line OVCAR-3 was obtained from the American Type Culture Collection (ATCC, Manassas, VA, USA) and cultured according to the manufacturer’s recommendations. Cells were maintained in RPMI-1640 medium (PanEco, Gorki Leninskiye, Moscow Region, Russia; C330p) supplemented with 20% heat-inactivated fetal bovine serum (HI-FBS; Gibco, 16140-071; Thermo Fisher Scientific, Grand Island, NY, USA), 2 mM L-glutamine (PanEco, F032), and bovine insulin at a final concentration of 0.01 mg/mL (PanEco, F060). Cultures were kept at 37 °C in a humidified atmosphere containing 5% CO_2_. To ensure consistency and reliability of the results, all experiments were performed using cells up to passage 15. During routine propagation, cells were maintained at a density of approximately 20,000 cells/cm^2^ and passaged twice weekly.

### 4.2. Compound Library and Experimental Design

A total of 54 commercially available small molecules were used in this study. The full list of compounds and their concise mechanism-of-action annotations is provided in Supplementary Table S1. The compounds were included exclusively for in vitro evaluation of their effects on OVCAR-3 cells and for testing the ability of the Cell Painting assay to distinguish biologically meaningful phenotypic perturbations associated with different mechanisms of action. The compound panel was divided into two cohorts of 27 molecules each to facilitate plate organization and experimental handling.

Cells were seeded into 384-well plates (Corning 3701; Corning Incorporated, Corning, NY, USA) using a multichannel pipette. Two seeding densities were evaluated during assay establishment. For experiments involving 2-day compound incubation, cells were seeded at 3000 cells per well, whereas for experiments with 8-day incubation, cells were seeded at 500 cells per well. This approach was chosen to account for differences in cell proliferation over time and to avoid excessive confluence after prolonged exposure, which could negatively affect segmentation quality and morphological profiling. For assay plates, cells were seeded in 20 µL of medium containing 10% FBS and no insulin, and were allowed to attach for 24 h at 37 °C in 5% CO_2_. Compounds were then added in an additional 20 µL of RPMI-1640 without FBS or insulin, resulting in a final well volume of 40 µL and a final FBS concentration of 5%. This treatment scheme was applied uniformly to all compound-treated and control wells in order to reduce proliferative activity during the exposure period, particularly under prolonged incubation conditions.

All compounds were initially prepared as 10 mM stocks in DMSO (Biolot, Saint Petersburg, Russia). Preliminary concentration-tolerance testing was performed using final compound concentrations of 1000, 100, 10, and 1 nM, with 0.1 nM additionally evaluated for highly cytotoxic compounds. Cell abundance was assessed by Hoechst nuclear staining and automated nuclear counting using an EnSight multimode plate reader (PerkinElmer, Waltham, MA, USA). Concentrations reducing nuclear counts below approximately 50% of the untreated control were considered excessively cytotoxic, and a lower concentration was used in subsequent experiments. Concentration selection was based primarily on limiting excessive loss of cell abundance rather than on maximizing or equalizing the Cell Painting morphological response. Actual concentrations used in each primary Cell Painting experiment are reported in Supplementary Table S1. For 17 compounds, concentrations differed between the 2-day and 8-day conditions; within each exposure condition, the concentration was constant across the primary biological replicates. Sixteen wells per plate contained cells but received neither compound nor DMSO and were excluded from compound-level analyses.

DMSO vehicle-control wells were prepared by a 1:100 dilution of DMSO in culture medium, followed by transfer of 20 µL into 480 µL medium and subsequent 1:1 dilution upon addition of 20 µL to 20 µL of medium in the assay plate, resulting in a final DMSO concentration of 0.02% (*v*/*v*). Because compound-treated wells were prepared from 10 mM DMSO stocks and different final compound concentrations were used, residual DMSO concentrations in compound-treated wells were not constant and ranged from approximately 0.000001% to 0.01% (*v*/*v*). The vehicle-control DMSO concentration was equal to or greater than that present in all compound-treated wells.

Within each biological replicate, each compound was dispensed into eight separate wells across different regions of the 384-well plate according to a manually designed randomization scheme. This strategy reduced positional bias while remaining practical for manual handling and minimizing pipetting errors. The plate layout is shown in Supplementary Figure S1.

The 2-day condition was performed in two independent biological replicates, whereas the 8-day condition was initially performed in three independent biological replicates. Biological replicates were defined as independent experimental runs performed on separate experimental days using separately cultured OVCAR-3 cell preparations. Prior to downstream clustering, replicate-level concordance was evaluated using Pearson correlations between compound-level morphological profiles. One biological replicate showed substantially lower concordance with the other two. Subsequent examination of experimental records showed that this replicate had been generated from cells harvested from an approximately 100% confluent source flask, whereas the two concordant replicates originated from cultures maintained at no more than approximately 70% confluence. The replicate was therefore excluded from the primary analysis as a post hoc culture-condition outlier. The decision to exclude this replicate was based on the documented source-culture condition together with its reduced concordance with the other biological replicates, and not on the resulting cluster assignment. To assess the impact of this decision, the full dataset including all three 8-day biological replicates was processed through the same scaling, PCA, exposure-duration selection, and Leiden clustering workflow. Agreement between the primary and full-replicate solutions was quantified using the adjusted Rand index for shared profiles.

### 4.3. Compound Incubation

Following compound addition, cells were maintained at 37 °C in 5% CO_2_ under the assay-medium conditions described above. Two treatment durations were investigated, namely 2 days and 8 days, in order to compare short-term and prolonged responses to chemical perturbation. These two incubation settings were subsequently incorporated into the computational workflow, allowing separate and integrated analyses of treatment-duration-dependent phenotypes.

No medium replacement was performed during either treatment period. To reduce evaporation and peripheral-well effects, the outer wells of the 384-well plates were filled with water and were not used for experimental samples.

### 4.4. Cell Painting Staining Protocol

The Cell Painting staining procedure was adapted for OVCAR-3 cells using a multiplex set of PhenoVue dyes (Revvity, Waltham, MA, USA). Two staining solutions were prepared. Solution 1 consisted of RPMI medium without FBS supplemented with PhenoVue 641 Mitochondrial Stain. Solution 2 contained 1× diluent, 0.1% Triton X-100 (Sigma-Aldrich, St. Louis, MO, USA), PhenoVue Fluor Hoechst 33342 Nuclear Stain, PhenoVue 512 Nucleic Acid Stain, PhenoVue Fluor 488–Concanavalin A, PhenoVue Fluor 555–WGA, and PhenoVue Fluor 568–Phalloidin. All PhenoVue reagents were obtained from Revvity (Waltham, MA, USA) and prepared according to the manufacturer’s recommendations. The dyes and corresponding cellular structures are summarized in Table 1. 

For mitochondrial staining, part of the culture medium was removed and replaced with Solution 1, followed by incubation for 30 min at 37 °C in the dark. Cells were then fixed with BD Cytofix (BD Biosciences, San Jose, CA, USA) for 20 min at room temperature protected from light. After fixation, wells were washed twice with HBSS (PanEco, Gorki Leninskiye, Moscow Region, Russia). Permeabilization and multiplex staining were performed by adding Solution 2 and incubating for 30 min at room temperature in the dark. Finally, cells were washed twice with HBSS. For temporary storage at 4 °C before imaging, wells were filled with HBSS containing 0.05% sodium azide (Sigma-Aldrich, St. Louis, MO, USA). 

### 4.5. Image Acquisition

Images were acquired using the CellInsight CX7 LED Pro High-Content Screening Platform (Thermo Fisher Scientific, Waltham, MA, USA) with a 20× objective. Exposure settings were optimized manually for each fluorescence channel to ensure adequate signal intensity while minimizing saturation. The final exposure times were 0.013 s for Hoechst, 0.015 s for the phalloidin/WGA channel, 0.035 s for the mitochondrial channel, 0.05 s for the endoplasmic reticulum-related channel, and 0.025 s for the nucleic acid channel. Imaging was performed in widefield mode using 2 × 2 binning with fixed exposure settings throughout the experiment. Background correction was applied to all fluorescence channels. Representative multichannel images of compound-treated OVCAR-3 cells acquired under these conditions are presented in Supplementary Figure S2.

Nine fields were acquired per well. Thus, each compound contributed up to 72 image sets per biological replicate (8 wells × 9 fields) and 288 image sets across the four primary 2-day/8-day biological-replicate datasets before QC exclusions.

### 4.6. Image Processing and Feature Extraction

Raw images were processed in CellProfiler version 4.2.8 using a workflow consisting of illumination correction, image quality control, object segmentation, and extraction of morphological features. The image analysis procedure followed the protocol described by Garcia-Fossa et al. [67], with modifications adapted to the OVCAR-3 staining panel and imaging conditions.

Image quality was assessed using CellProfiler image-quality metrics. Image sharpness was monitored using the PowerLogLogSlope metric calculated for the mitochondrial channel, and image sets with values below −2.8 were classified as blurry and excluded from further processing. Image sets containing fewer than 20 detected nuclei were also excluded.

Nuclei were segmented from the DNA channel, whole-cell boundaries were identified using the RNA channel, and cytoplasmic regions were defined as the cell area excluding the nucleus. Objects touching the image border were removed. CellProfiler then extracted quantitative descriptors, including intensity, texture, granularity, size and shape, radial intensity distribution, spatial relationships, and colocalization measurements.

### 4.7. Correlation Analysis of Morphological Features

To examine relationships among the extracted morphological profiles, Pearson correlation analysis was performed and visualized in Morpheus https://software.broadinstitute.org/morpheus/ (accessed on 28 February 2026). The morphological feature table was uploaded to the platform, and pairwise Pearson correlation coefficients were calculated. The resulting heatmap was used to visualize the overall correlation structure of the dataset.

### 4.8. Data Preprocessing and Clustering Analysis in Python

Further analysis was performed in Python 3.14.3 using pandas 3.0.1, NumPy 2.4.2, SciPy 1.17.1, scikit-learn 1.8.0, Scanpy 1.12.0, and Matplotlib 3.10.8. Metadata were harmonized and compound labels were standardized to support interpretation and visualization. CellProfiler-derived measurements were first aggregated to the well level using the median within each plate. Well-level morphological profiles were then normalized separately within each plate relative to the corresponding DMSO control wells using the mad_robustize method implemented in pycytominer version 1.4.0. DMSO wells therefore served as the negative-control population for plate-wise normalization.

Following well-level aggregation, the analysis table contained 5272 quantitative morphological feature columns in addition to metadata. Feature selection using pycytominer version 1.4.0 reduced this set to 236 features and included correlation-threshold filtering, variance-threshold filtering, removal of features containing missing values, application of the Cell Painting blocklist, and removal of outlier features.

Before principal component analysis, these selected features were additionally standardized to zero mean and unit variance using StandardScaler (version 1.7.2).

Dimensionality reduction was performed using principal component analysis (PCA), with the number of retained principal components treated as a tunable parameter. For each evaluated number of principal components, a single PCA model was fitted to the full standardized feature matrix containing both treatment-duration conditions. The resulting PCA representation was subsequently divided into the short- and long-treatment datasets, ensuring that profiles from both conditions were represented in the same coordinate space.

Morphological profiles were analyzed under the assumption that compounds producing similar cellular responses would generate similar high-dimensional feature signatures. Unsupervised clustering was therefore used to identify groups of compounds associated with shared phenotypic states without imposing predefined mechanism-of-action categories.

Three related datasets were considered for downstream analysis. The short dataset contained profiles obtained after the shorter incubation period, whereas the long dataset contained profiles generated after prolonged incubation. For each PCA and distance-metric combination evaluated during grid search, a corresponding combined dataset was generated. For each compound, the mean pairwise distance among its replicate-well profiles in PCA space was calculated separately for the short- and long-treatment conditions. The condition with the lower mean pairwise distance was retained for inclusion in the combined dataset. This criterion was used to select the treatment condition showing greater within-compound phenotypic consistency.

Before clustering, PCA profiles were aggregated at the level of each compound and biological replicate using the median across replicate wells. In the primary Combined dataset, each compound was represented by two biological-replicate profiles. One Docetaxel biological-replicate profile was unavailable in the 8-day-only dataset.

To determine whether individual compounds produced morphological responses distinguishable from vehicle controls, activity was assessed separately within each primary dataset using the DMSO-normalized morphological profiles. For each compound, the Euclidean distance between the mean compound profile and the corresponding DMSO centroid was calculated. A DMSO-derived null distribution was generated by repeatedly sampling DMSO wells, without replacement, using the same number of wells as available for the tested compound. The mean profile of the sampled subset was treated as a pseudo-compound and compared with the remaining DMSO wells. This procedure was repeated 5000 times. A compound was classified as morphologically active when its observed distance exceeded the 95th percentile of the corresponding DMSO-derived null distribution. Reproducible activity at a given exposure duration was defined as activity in both independent biological replicates.

The primary clustering method was Leiden clustering. To evaluate whether the graph-based approach provided an advantage over more conventional methods, Leiden clustering was compared with k-means clustering and agglomerative hierarchical clustering. Leiden clustering was implemented in scanpy using a k-nearest-neighbor graph constructed from PCA coordinates. K-means and agglomerative clustering were implemented in scikit-learn.

Grid search evaluated 50, 55, 60, 65, and 70 principal components and Euclidean, cosine, and correlation distance metrics. For Leiden clustering, the number of nearest neighbors ranged from 5 to 30 in increments of 5 and the resolution parameter ranged from 0.2 to 3.0 in increments of 0.2. For k-means and agglomerative clustering, the number of clusters ranged from 4 to 8. Parameter combinations producing fewer than five clusters were excluded from model selection. The silhouette score was used as the primary optimization criterion. A random state of 42 was specified for stochastic procedures where applicable.

The search spaces were not identical across clustering algorithms and therefore the maximum silhouette values were treated as descriptive rather than as a formal head-to-head test of algorithm superiority. In addition, silhouette values calculated using different distance metrics were not assumed to be directly comparable on an absolute scale. Configurations yielding fewer than five clusters were excluded to avoid highly coarse partitions; this threshold was heuristic and was not biologically prespecified.

To estimate the dispersion of silhouette values for the selected clustering solutions, 1000 repeated compound-level subsamples were generated. In each iteration, 80% of compounds (43 of 54) were sampled while retaining both biological-replicate profiles for each selected compound. Silhouette scores were recalculated using the fixed final cluster assignments without re-optimizing or re-fitting the clustering models.

Associations between Leiden clusters and broad mechanism-of-action classes were evaluated using one-sided Fisher exact tests. Split compounds were excluded from this analysis, and mechanism classes represented by at least two compounds were considered. The resulting cluster-by-mechanism tests were corrected jointly for multiple comparisons using the Benjamini–Hochberg false-discovery-rate procedure.

Replicate consistency was additionally quantified using a percent-replicating analysis in the 50-component PCA representation with cosine distance. Statistical significance was assessed against a shuffled-label null distribution.

Mechanism-of-action retrieval was quantified using mean average precision (mAP) after aggregating biological replicates to one median profile per compound. Compounds belonging to mechanism classes represented by at least two compounds were included. A null distribution was generated by 5000 permutations of the mechanism labels.

For k-means clustering, the standard Euclidean k-means objective was used; the evaluated distance metric was used for construction of the corresponding combined dataset and calculation of the silhouette score. For agglomerative clustering, Ward linkage was used with Euclidean distance and average linkage with non-Euclidean distance metrics.

As an additional measure of reproducibility across biological replicates, we calculated the number of split compounds. After median aggregation across replicate wells, each compound was represented by one profile per biological replicate. A compound was considered split when its two biological-replicate profiles were assigned to different clusters. UMAP was used exclusively for visualization and was not used for distance calculation, clustering, or model selection [68]. As a diagnostic for residual experimental structure, the Combined UMAP was additionally visualized according to compound cohort, plate, biological replicate, and selected exposure duration.

The analytical workflow was designed to characterize the organization of drug-induced phenotypic responses in OVCAR-3 cells rather than to evaluate predefined biological classes. Clustering results were interpreted as morphology-based phenotypic states that could subsequently be compared with known mechanisms of action.

As an additional diagnostic analysis, DMSO control profiles were aggregated at the dataset level and projected together with the 108 drug profiles representing 54 compounds × two biological replicates. Eight DMSO profiles were included, corresponding to two exposure durations, two compound cohorts, and two biological replicates. The same PCA representation and cosine-neighbor graph settings used for the primary Leiden analysis were applied. This analysis was used to evaluate the location of vehicle controls relative to drug-induced profiles and the stability of compound cluster assignments after inclusion of DMSO controls.

To evaluate the influence of paired biological-replicate profiles on the silhouette statistic, the two primary biological-replicate profiles for each compound were additionally median-aggregated to a single compound-level profile, and the silhouette score was recalculated at the compound level.

## 5. Conclusions

Cell Painting profiling of 54 anticancer compounds in OVCAR-3 cells identified mechanism-associated morphological patterns, including reproducible organization of several microtubule- and DNA-damage-related compounds. However, same-mechanism compounds were not uniformly co-clustered, demonstrating that morphological similarity does not provide a one-to-one representation of annotated molecular mechanisms. The identified phenotypic clusters should therefore be interpreted as reflecting both compound-induced cellular responses and the experimental exposure context, including treatment duration and associated differences in seeding density. These findings support the use of Cell Painting for identifying mechanism-associated phenotypic patterns while highlighting its limited resolution for grouping compounds solely according to molecular target annotation.

## Figures and Tables

**Table 1:** Fluorescent dyes included in the Cell Painting assay and the corresponding cellular structures visualized in OVCAR-3 cells.

Dye	Cellular Compartment
PhenoVue 641 Mitochondrial Stain	Mitochondria
PhenoVue Fluor 555—WGA	Golgi apparatus, plasma membrane
PhenoVue Fluor 488—Concanavalin A	endoplasmic reticulum
PhenoVue Fluor 568—Phalloidin	Actin, cytoskeleton
PhenoVue Fluor Hoechst 33342 Nuclear Stain	Nucleus
PhenoVue 512 Nucleic Acid Stain	Nucleic acids

## Data Availability

Processed Cell Painting data, the CellProfiler analysis pipeline, Python analysis code, compound concentration information, aggregated feature matrices, Supplementary Table S1, and fully labeled vector versions of the correlation heatmaps are publicly available in Zenodo at https://doi.org/10.5281/zenodo.22958654. Raw microscopy images are not publicly available due to institutional data-management restrictions.

## Supplementary Materials

The following supporting information can be downloaded at: https://www.mdpi.com/article/10.3390/ijms27198791/s1.

## Abbreviations

The following abbreviations are used in this manuscript:

PCA	Principal Component Analysis
UMAP	Uniform Manifold Approximation and Projection
EGFR	Epidermal Growth Factor Receptor
BRAF	v-Raf Murine Sarcoma Viral Oncogene Homolog B1
MAPK	Mitogen-Activated Protein Kinase
PI3Kα	Phosphoinositide 3-Kinase Alpha
mTOR	Mechanistic Target of Rapamycin
VEGFR	Vascular Endothelial Growth Factor Receptor
TRK	Tropomyosin Receptor Kinase
ROS1	ROS Proto-Oncogene 1, Receptor Tyrosine Kinase
ALK	Anaplastic Lymphoma Kinase
MET	Mesenchymal–Epithelial Transition Factor
HER2	Human Epidermal Growth Factor Receptor 2
RET	Rearranged during Transfection
DHFR	Dihydrofolate Reductase
PARP	Poly(ADP-ribose) Polymerase
GnRH	Gonadotropin-Releasing Hormone
CDK4/6	Cyclin-Dependent Kinases 4 and 6
