# Supplementary material for: Cell Painting Identifies Phenotypic Clusters Associated with Anticancer Drug Responses in OVCAR-3 Ovarian Cancer Cells
Source: Int J Mol Sci. 2026 Sep 30;27(19):8791. doi: 10.3390/ijms27198791 (PMC13664869; doi:10.3390/ijms27198791)
Supplement: Supplementary file 1 [file ijms-27-08791-s001.zip › ijms-SUPP.pdf]

Supplementary

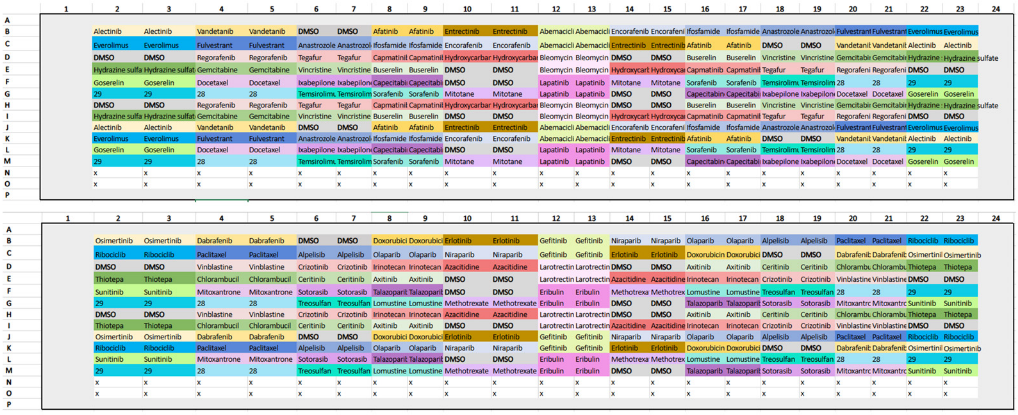

	1	2	3	4	5	6	7	8	9	10	11	12	13	14	15	16	17	18	19	20	21	22	23	24
A																								
B		Alectinib	Alectinib	Vandetanib	Vandetanib	DMSO	DMSO	Afatinib	Afatinib	Entrectinib	Entrectinib	Abemacicli	Abemacicli	Encorafenib	Encorafeni	Ifosfamide	Ifosfamide	Anastrozole	Anastrozol	Fulvestrant	Fulvestrant	Everolimus	Everolimus	
C		Everolimus	Everolimus	Fulvestrant	Fulvestrant	Anastrozole	Anastrozol	Ifosfamide	Ifosfamide	Encorafenib	Encorafenib	Abemacicli	Abemacicli	Entrectinib	Entrectinib	Afatinib	Afatinib	DMSO	DMSO	Vandetanib	Vandetanib	Alectinib	Alectinib	
D		DMSO	DMSO	Regorafenib	Regorafenib	Tegafur	Tegafur	Capmatinib	Capmatinib	Hydroxycarbar	Hydroxycarbar	Bleomycin	Bleomycin	DMSO	DMSO	Buserelin	Buserelin	Vincristine	Vincristine	Gemcitabi	Gemcitabi	Hydrazine	Hydrazine sulfate	
E		Hydrazine sulfa	Hydrazine sulfat	Gemcitabine	Gemcitabine	Vincristine	Vincristine	Buserelin	Buserelin	DMSO	DMSO	Bleomycin	Bleomycin	Hydroxycarb	Hydroxycar	Capmatinib	Capmatinib	Tegafur	Tegafur	Regorafeni	Regorafeni	DMSO	DMSO	
F		Goserelin	Goserelin	Docetaxel	Docetaxel	Ixabepilone	Ixabepilon	Capecitabi	Capecitabi	DMSO	DMSO	Lapatinib	Lapatinib	Mitotane	Mitotane	Sorafenib	Sorafenib	Temsirolimu	Temsirolim	28	28	29	29	
G		29	29	28	28	Temsirolimu	Temsirolim	Sorafenib	Sorafenib	Mitotane	Mitotane	Lapatinib	Lapatinib	DMSO	DMSO	Capecitabin	Capecitabi	Ixabepilone	Ixabepilon	Docetaxel	Docetaxel	Goserelin	Goserelin	
H		DMSO	DMSO	Regorafenib	Regorafenib	Tegafur	Tegafur	Capmatinib	Capmatinib	Hydroxycarbar	Hydroxycarbar	Bleomycin	Bleomycin	DMSO	DMSO	Buserelin	Buserelin	Vincristine	Vincristine	Gemcitabi	Gemcitabi	Hydrazine	Hydrazine sulfate	
I		Hydrazine sulfa	Hydrazine sulfat	Gemcitabine	Gemcitabine	Vincristine	Vincristine	Buserelin	Buserelin	DMSO	DMSO	Bleomycin	Bleomycin	Hydroxycarb	Hydroxycar	Capmatinib	Capmatinib	Tegafur	Tegafur	Regorafeni	Regorafeni	DMSO	DMSO	
J		Alectinib	Alectinib	Vandetanib	Vandetanib	DMSO	DMSO	Afatinib	Afatinib	Entrectinib	Entrectinib	Abemacicli	Abemacicli	Encorafenib	Encorafeni	Ifosfamide	Ifosfamide	Anastrozole	Anastrozol	Fulvestrant	Fulvestrant	Everolimus	Everolimus	
K		Everolimus	Everolimus	Fulvestrant	Fulvestrant	Anastrozole	Anastrozol	Ifosfamide	Ifosfamide	Encorafenib	Encorafenib	Abemacicli	Abemacicli	Entrectinib	Entrectinib	Afatinib	Afatinib	DMSO	DMSO	Vandetanib	Vandetanib	Alectinib	Alectinib	
L		Goserelin	Goserelin	Docetaxel	Docetaxel	Ixabepilone	Ixabepilon	Capecitabi	Capecitabi	DMSO	DMSO	Lapatinib	Lapatinib	Mitotane	Mitotane	Sorafenib	Sorafenib	Temsirolimu	Temsirolim	28	28	29	29	
M		29	29	28	28	Temsirolimu	Temsirolim	Sorafenib	Sorafenib	Mitotane	Mitotane	Lapatinib	Lapatinib	DMSO	DMSO	Capecitabin	Capecitabi	Ixabepilone	Ixabepilon	Docetaxel	Docetaxel	Goserelin	Goserelin	
N		x	x	x	x	x	x	x	x	x	x	x	x	x	x	x	x	x	x	x	x	x	x	
O		x	x	x	x	x	x	x	x	x	x	x	x	x	x	x	x	x	x	x	x	x	x	
P																								

	1	2	3	4	5	6	7	8	9	10	11	12	13	14	15	16	17	18	19	20	21	22	23	24
A																								
B		Osimertinib	Osimertinib	Dabrafenib	Dabrafenib	DMSO	DMSO	Doxorubici	Doxorubici	Erlotinib	Erlotinib	Gefitinib	Gefitinib	Niraparib	Niraparib	Olaparib	Olaparib	Alpelisib	Alpelisib	Paclitaxel	Paclitaxel	Ribociclib	Ribociclib	
C		Ribociclib	Ribociclib	Paclitaxel	Paclitaxel	Alpelisib	Alpelisib	Olaparib	Olaparib	Niraparib	Niraparib	Gefitinib	Gefitinib	Erlotinib	Erlotinib	Doxorubicin	Doxorubici	DMSO	DMSO	Dabrafenib	Dabrafenib	Osimertinib	Osimertinib	
D		DMSO	DMSO	Vinblastine	Vinblastine	Crizotinib	Crizotinib	Irinotecan	Irinotecan	Azacitidine	Azacitidine	Larotrectin	Larotrectin	DMSO	DMSO	Axitinib	Axitinib	Ceritinib	Ceritinib	Chlorambu	Chlorambu	Thiotepa	Thiotepa	
E		Thiotepa	Thiotepa	Chlorambucil	Chlorambucil	Ceritinib	Ceritinib	Axitinib	Axitinib	DMSO	DMSO	Larotrectin	Larotrectin	Azacitidine	Azacitidine	Irinotecan	Irinotecan	Crizotinib	Crizotinib	Vinblastine	Vinblastine	DMSO	DMSO	
F		Sunitinib	Sunitinib	Mitoxantrone	Mitoxantrone	Sotorasib	Sotorasib	Talazoparib	Talazoparib	DMSO	DMSO	Eribulin	Eribulin	Methotrexa	Methotrex	Lomustine	Lomustine	Treosulfan	Treosulfan	28	28	29	29	
G		29	29	28	28	Treosulfan	Treosulfan	Lomustine	Lomustine	Methotrexate	Methotrexate	Eribulin	Eribulin	DMSO	DMSO	Talazoparib	Talazoparib	Sotorasib	Sotorasib	Mitoxantrc	Mitoxantrc	Sunitinib	Sunitinib	
H		DMSO	DMSO	Vinblastine	Vinblastine	Crizotinib	Crizotinib	Irinotecan	Irinotecan	Azacitidine	Azacitidine	Larotrectin	Larotrectin	DMSO	DMSO	Axitinib	Axitinib	Ceritinib	Ceritinib	Chlorambu	Chlorambu	Thiotepa	Thiotepa	
I		Thiotepa	Thiotepa	Chlorambucil	Chlorambucil	Ceritinib	Ceritinib	Axitinib	Axitinib	DMSO	DMSO	Larotrectin	Larotrectin	Azacitidine	Azacitidine	Irinotecan	Irinotecan	Crizotinib	Crizotinib	Vinblastine	Vinblastine	DMSO	DMSO	
J		Osimertinib	Osimertinib	Dabrafenib	Dabrafenib	DMSO	DMSO	Doxorubici	Doxorubici	Erlotinib	Erlotinib	Gefitinib	Gefitinib	Niraparib	Niraparib	Olaparib	Olaparib	Alpelisib	Alpelisib	Paclitaxel	Paclitaxel	Ribociclib	Ribociclib	
K		Ribociclib	Ribociclib	Paclitaxel	Paclitaxel	Alpelisib	Alpelisib	Olaparib	Olaparib	Niraparib	Niraparib	Gefitinib	Gefitinib	Erlotinib	Erlotinib	Doxorubicin	Doxorubici	DMSO	DMSO	Dabrafenib	Dabrafenib	Osimertinib	Osimertinib	
L		Sunitinib	Sunitinib	Mitoxantrone	Mitoxantrone	Sotorasib	Sotorasib	Talazoparib	Talazoparib	DMSO	DMSO	Eribulin	Eribulin	Methotrexa	Methotrex	Lomustine	Lomustine	Treosulfan	Treosulfan	28	28	29	29	
M		29	29	28	28	Treosulfan	Treosulfan	Lomustine	Lomustine	Methotrexate	Methotrexate	Eribulin	Eribulin	DMSO	DMSO	Talazoparib	Talazoparib	Sotorasib	Sotorasib	Mitoxantrc	Mitoxantrc	Sunitinib	Sunitinib	
N		x	x	x	x	x	x	x	x	x	x	x	x	x	x	x	x	x	x	x	x	x	x	
O		x	x	x	x	x	x	x	x	x	x	x	x	x	x	x	x	x	x	x	x	x	x	
P																								

Figure S1. Composition of compound cohorts in the OVCAR-3 Cell Painting assay.

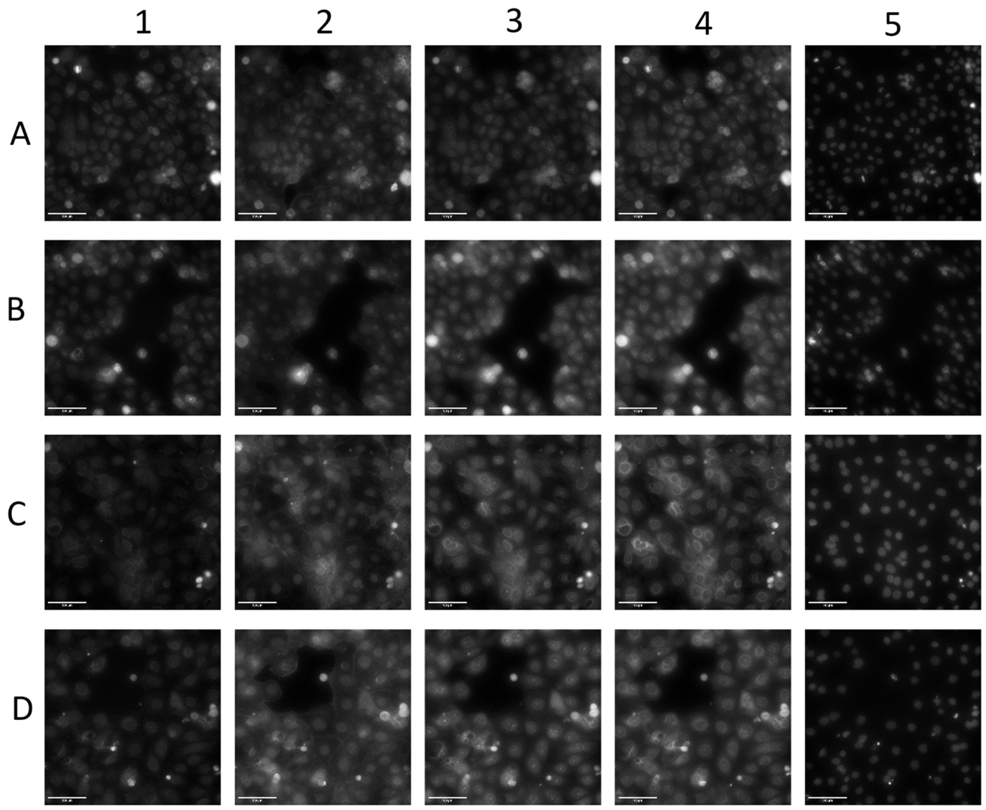

Figure S2. Representative Cell Painting images of OVCAR-3 cells after 2-day treatment with selected compounds, acquired in individual fluorescence channels. (A) Bleomycin (B) Paclitaxel (C) Erlotinib (D) Irinotecan. The staining set visualized the following cellular compartments: mitochondria (PhenoVue 641 Mitochondrial Stain), Golgi apparatus and plasma membrane (PhenoVue Fluor 555–WGA), nucleic acids (PhenoVue 512 Nucleic Acid Stain), actin cytoskeleton (PhenoVue Fluor 568–Phalloidin), endoplasmic reticulum (PhenoVue Fluor 488–Concanavalin A), and nucleus (PhenoVue Fluor Hoechst 33342 Nuclear Stain).

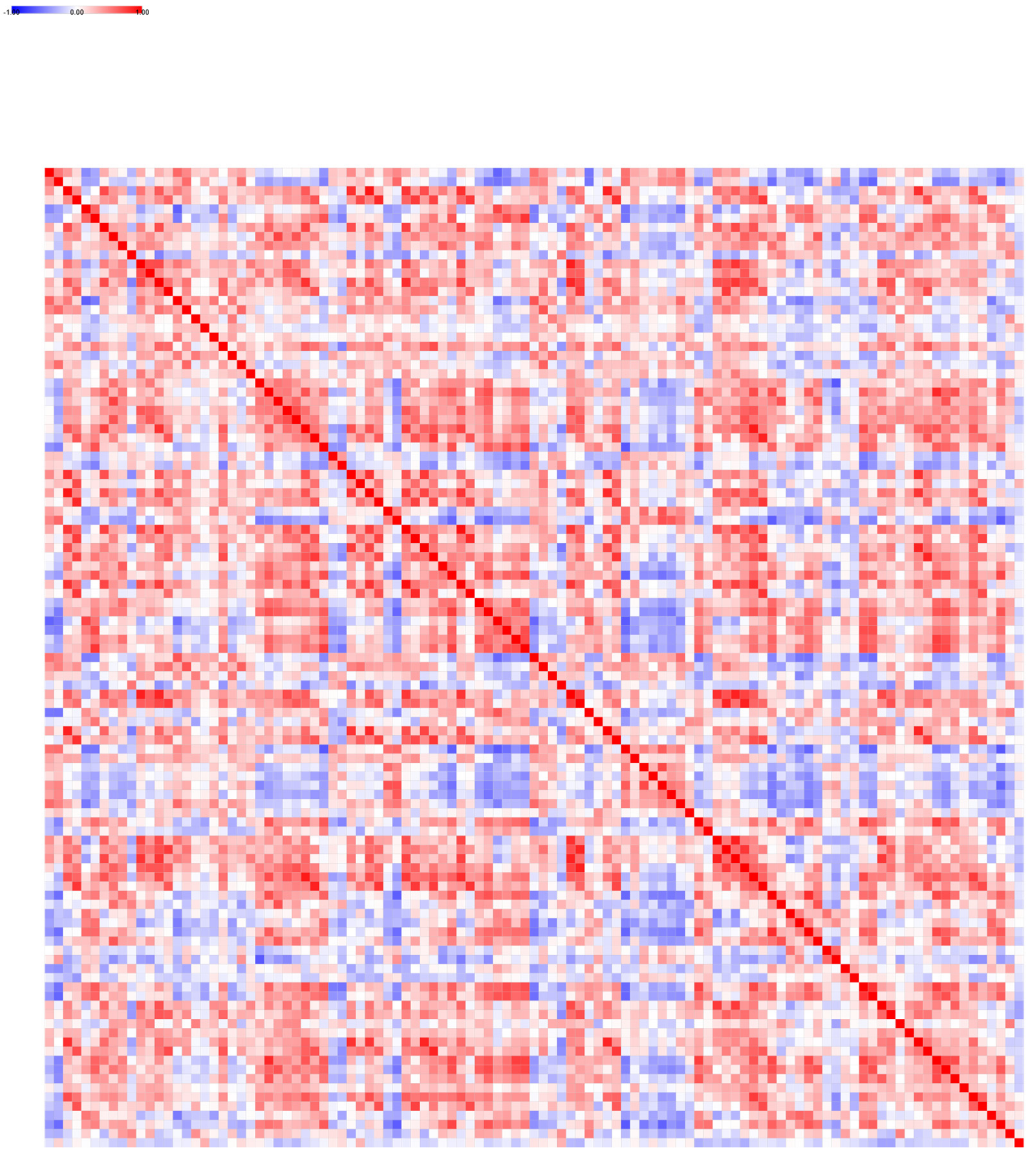

Figure S3. Pearson correlation heatmap of Cell Painting-derived morphological profiles after 8-day compound incubation. Axis labels were omitted for readability because of the large number of profiles. Profiles are displayed in the same order on both axes. The corresponding fully labeled vector heatmap is available in the public Zenodo repository (https://doi.org/10.5281/zenodo.22958654).

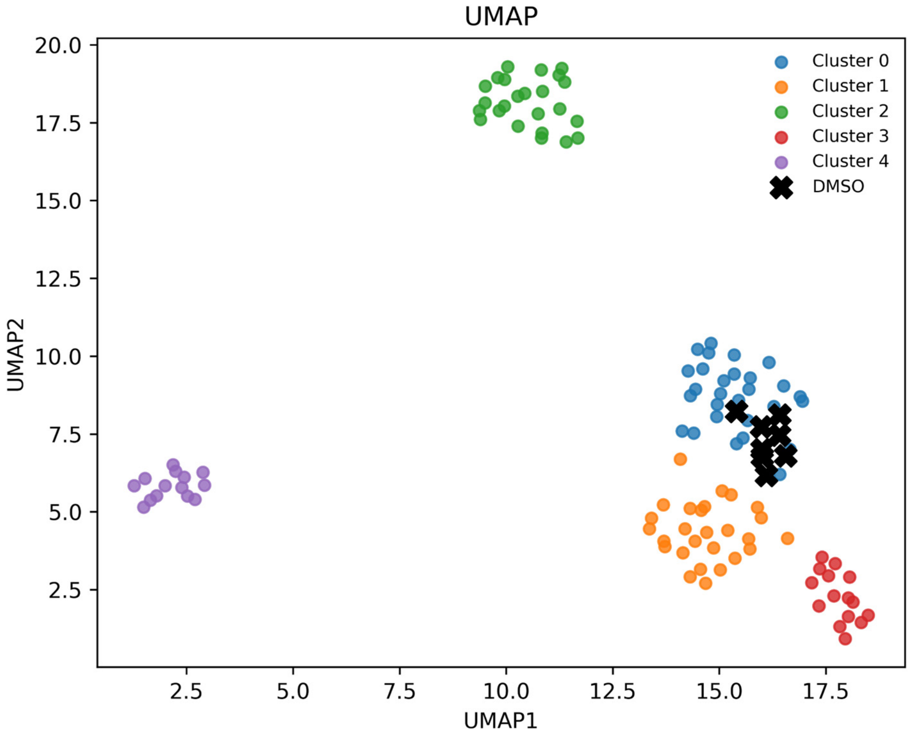

Figure S4. Diagnostic inclusion of DMSO control profiles in the OVCAR-3 Cell Painting phenotypic space. Eight dataset-level DMSO profiles, corresponding to two exposure durations, two compound cohorts, and two biological replicates, were projected together with 108 drug profiles representing 54 compounds × two biological replicates. DMSO controls formed a compact group, whereas drug cluster assignments remained highly consistent with the primary drug-only clustering solution.

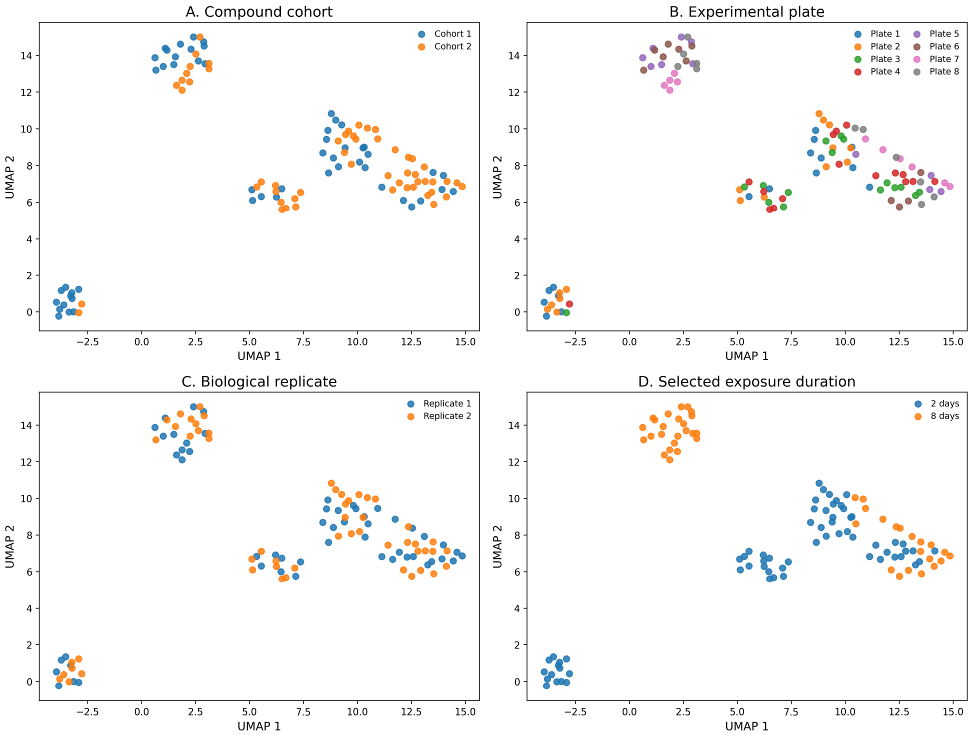

Figure S5. Diagnostic visualization of experimental structure in the Combined OVCAR-3 Cell Painting dataset. The UMAP representation was recolored according to (A) compound cohort, (B) experimental plate, (C) biological replicate, and (D) selected exposure duration. For clarity, the original cohorts 10 and 11 are labeled Cohort 1 and Cohort 2, respectively, and the original plate identifiers are simplified as Plate 1–8.

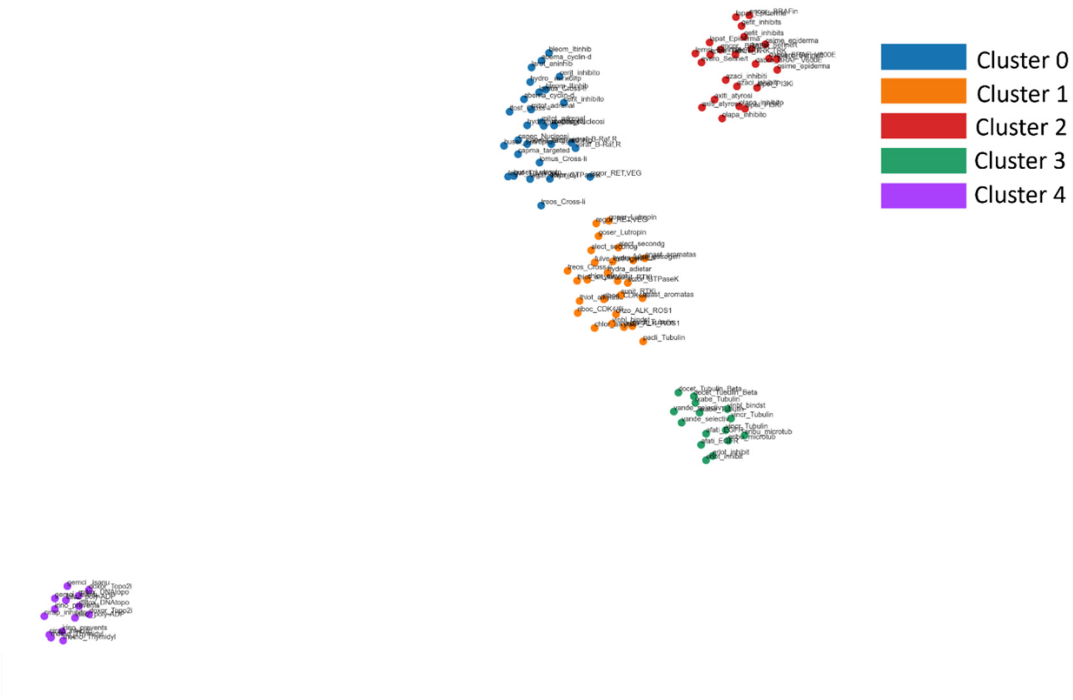

Figure S6. UMAP visualization of the combined OVCAR-3 Cell Painting dataset clustered with agglomerative clustering. The agglomerative clustering solution was generated using cosine distance with average linkage and 50 principal components, yielding 5 clusters with a silhouette score of 0.482 and 4 split compounds.

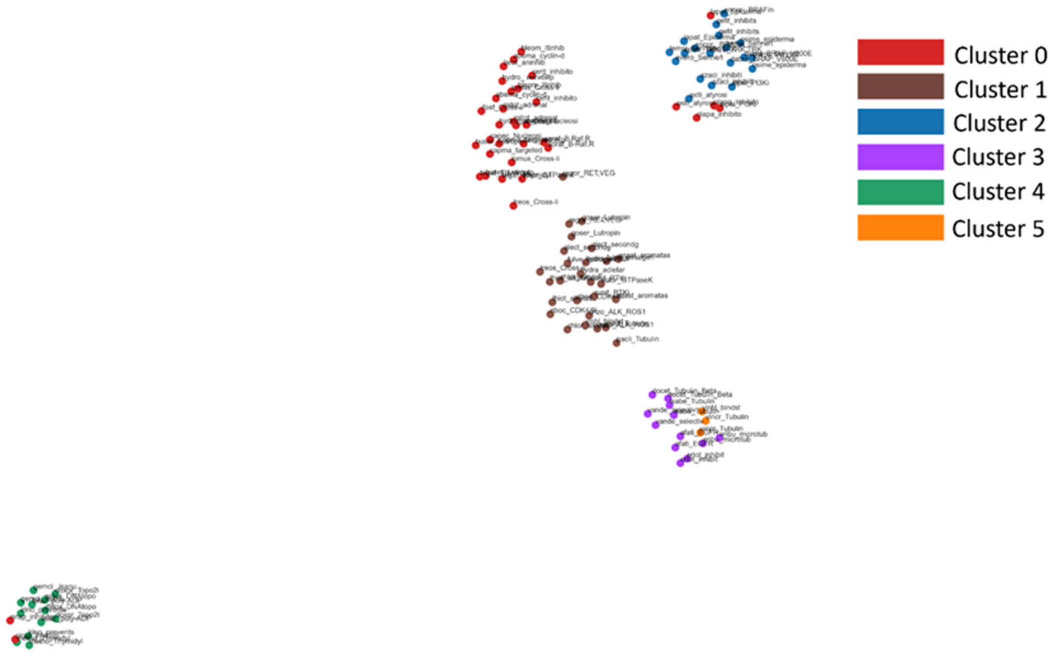

Figure S7. UMAP visualization of the combined OVCAR-3 Cell Painting dataset clustered with k-means. The combined dataset shown here was constructed using cosine distance and 50 principal components. K-means clustering yielded 6 clusters, with a cosine-based silhouette score of 0.408 and 6 split compounds.

Supplementary Table S2. Cluster–mechanism enrichment analysis using one-sided Fisher exact tests with Benjamini–Hochberg FDR correction. Notes: Compounds in cluster / cluster size indicates the number of compounds of the specified broad mechanism class among all compounds assigned to that cluster. Total compounds in class indicates the number of compounds of that class in the full enrichment dataset (n = 51 non-split compounds). Odds ratios >1 indicate over-representation. P values were obtained using one-sided Fisher exact tests and adjusted across all 45 cluster–mechanism comparisons using the Benjamini–Hochberg procedure. FDR-adjusted P < 0.05 was considered statistically significant. Split compounds were excluded from the analysis. Only broad mechanism classes represented by at least two compounds were tested.

Cluster	Broad mechanism class	Compounds in cluster / cluster size	Total compounds in class	Odds ratio	P value	FDR-adjusted P	FDR < 0.05
0	Alkylating agent	2/13	4	3.273	0.265774	1.000000	No
	Antimetabolite	3/13	5	5.400	0.097705	0.628104	No
	Cell cycle inhibitor	1/13	2	3.083	0.448627	1.000000	No
	DNA repair inhibitor	0/13	3	0.000	1.000000	1.000000	No
	Endocrine therapy	1/13	4	0.972	0.704622	1.000000	No
	Kinase inhibitor	4/13	19	0.681	0.812680	1.000000	No
	mTOR inhibitor	0/13	2	0.000	1.000000	1.000000	No
	Microtubule-targeting agent	0/13	5	0.000	1.000000	1.000000	No
	Topoisomerase inhibitor	0/13	3	0.000	1.000000	1.000000	No
1	Alkylating agent	2/12	4	3.700	0.232017	1.000000	No
	Antimetabolite	0/12	5	0.000	1.000000	1.000000	No
	Cell cycle inhibitor	1/12	2	3.455	0.418824	1.000000	No
	DNA repair inhibitor	0/12	3	0.000	1.000000	1.000000	No
	Endocrine therapy	3/12	4	12.667	0.036315	0.388235	No

	Kinase inhibitor	4/12	19	0.800	0.742442	1.000000	No
	mTOR inhibitor	0/12	2	0.000	1.000000	1.000000	No
	Microtubule-targeting agent	1/12	5	0.795	0.754899	1.000000	No
	Topoisomerase inhibitor	0/12	3	0.000	1.000000	1.000000	No
2	Alkylating agent	0/12	4	0.000	1.000000	1.000000	No
	Antimetabolite	0/12	5	0.000	1.000000	1.000000	No
	Cell cycle inhibitor	0/12	2	0.000	1.000000	1.000000	No
	DNA repair inhibitor	1/12	3	1.682	0.561152	1.000000	No
	Endocrine therapy	0/12	4	0.000	1.000000	1.000000	No
	Kinase inhibitor	8/12	19	5.091	0.020311	0.304662	No
	mTOR inhibitor	2/12	2	∞	0.051765	0.388235	No
	Microtubule-targeting agent	0/12	5	0.000	1.000000	1.000000	No
	Topoisomerase inhibitor	0/12	3	0.000	1.000000	1.000000	No
3	Alkylating agent	0/7	4	0.000	1.000000	1.000000	No
	Antimetabolite	0/7	5	0.000	1.000000	1.000000	No
	Cell cycle inhibitor	0/7	2	0.000	1.000000	1.000000	No
	DNA repair inhibitor	0/7	3	0.000	1.000000	1.000000	No
	Endocrine therapy	0/7	4	0.000	1.000000	1.000000	No
	Kinase inhibitor	3/7	19	1.313	0.524779	1.000000	No
	mTOR inhibitor	0/7	2	0.000	1.000000	1.000000	No
	Microtubule-	4/7	5	57.333	0.000665	0.029903	Yes

	targeting agent						
	Topoisomerase inhibitor	0/7	3	0.000	1.000000	1.000000	No
4	Alkylating agent	0/7	4	0.000	1.000000	1.000000	No
	Antimetabolite	2/7	5	5.467	0.133158	0.749011	No
	Cell cycle inhibitor	0/7	2	0.000	1.000000	1.000000	No
	DNA repair inhibitor	2/7	3	17.200	0.046050	0.388235	No
	Endocrine therapy	0/7	4	0.000	1.000000	1.000000	No
	Kinase inhibitor	0/7	19	0.000	1.000000	1.000000	No
	mTOR inhibitor	0/7	2	0.000	1.000000	1.000000	No
	Microtubule-targeting agent	0/7	5	0.000	1.000000	1.000000	No
	Topoisomerase inhibitor	3/7	3	∞	0.001681	0.037815	Yes

Supplementary Table S3. Sensitivity analyses of the Combined OVCAR-3 Cell Painting clustering solution. (A) Inclusion of the third 8-day biological replicate. (B) Exclusion of the 12 compounds without a reproducible morphological response distinguishable from DMSO at the Combined-selected duration. (C) Compound-level silhouette analysis after median aggregation of the two primary biological replicates.

Panel A. Inclusion of the third 8-day biological replicate			
Metric	Primary analysis	Full 3-replicate sensitivity	Notes
Compounds	54	54	Same compound panel
Biological-replicate profiles	108	131	31 compounds selected at 2 d contributed 2 profiles each; 23 compounds selected at 8 d contributed 3 profiles each
Selected 2-day compounds	32	31	Mitotane changed from 2 d to 8 d
Selected 8-day compounds	22	23	Mitotane changed from 2 d to 8 d
Leiden clusters	5	5	Fixed Leiden settings
Silhouette score	0.4856	0.4865	Cosine distance
Split compounds	3	4	Primary: Sotorasib, Treosulfan, Vinblastine; full analysis additionally split Regorafenib
Adjusted Rand index	Reference	0.9715	Primary vs full analysis, calculated for the 106 shared profiles
Compounds changing selected duration		1	Mitotane: 2 d → 8 d
Panel B. Exclusion of 12 compounds without reproducible DMSO departure at the selected duration			
Metric	Primary analysis	Exclude-12 sensitivity	Notes
Compounds	54	42	12 compounds removed
Biological-replicate profiles	108	84	Two primary biological-replicate profiles per retained compound
Selected 2-day compounds	32	25	Seven excluded compounds had 2 d selected
Selected 8-day compounds	22	17	Five excluded compounds had 8 d selected
Leiden clusters	5	5	Same fixed Leiden settings
Silhouette score	0.4856	0.5081	Cosine distance; the original 50-PC representation was retained
Split compounds	3	1	Vinblastine was the only split compound after exclusion
Adjusted Rand index	Reference	1	Original vs exclude-12 clustering for the 84 retained profiles; cluster labels differed only by permutation
Panel C. Compound-level silhouette after median aggregation of the two primary biological replicates			

Metric	Primary profile-level analysis	Compound-level aggregation	Notes
Profiles	108	54	One median profile per compound in the compound-level analysis
Leiden clusters	5	5	Same five-cluster organization
Silhouette score	0.4856	0.5446	Cluster separation remained evident after collapsing the two primary biological replicates to one profile per compound

Supplementary Table S4. Image-quality control summary by compound and control condition. Note: Untreated wells contained cells but received neither test compound nor DMSO and were not included in the compound-level analysis.

Compound / annotation	Total image sets	Blurry image sets	Image sets with <20 nuclei	Excluded image sets	Median nuclei per image	Excluded image sets (%)
Vincristine	288	29	5	34	95.5	11.81%
Tegafur	288	28	1	29	176.5	10.07%
Crizotinib	288	22	1	23	157.0	7.99%
Vandetanib	288	16	5	21	112.0	7.29%
Osimertinib	288	9	10	19	87.5	6.60%
Mitoxantrone	288	16	0	16	104.0	5.56%
Chlorambucil	288	13	2	15	148.0	5.21%
Temsirolimus	288	12	2	14	113.0	4.86%
Sotorasib	288	14	0	14	170.0	4.86%
Dabrafenib	288	6	6	12	104.0	4.17%
Everolimus	288	3	8	11	101.0	3.82%
Ixabepilone	288	9	2	11	146.0	3.82%
Ceritinib	288	7	4	11	154.0	3.82%
Gefitinib	288	7	3	10	110.0	3.47%
Ribociclib	288	6	4	10	161.0	3.47%
Vinblastine	288	9	1	10	127.0	3.47%
Azacitidine	288	6	4	10	116.0	3.47%
Eribulin	288	10	0	10	147.0	3.47%
Afatinib	288	5	4	9	102.0	3.13%
Entrectinib	288	4	5	9	115.0	3.13%
Fulvestrant	288	8	1	9	160.0	3.13%
Gemcitabine	288	5	4	9	105.0	3.13%
Erlotinib	288	3	6	9	118.0	3.13%
Doxorubicin	288	8	0	8	100.0	2.78%
Alpelisib	288	7	1	8	125.0	2.78%
Irinotecan	288	5	3	8	91.0	2.78%
Larotrectinib	288	7	1	8	158.5	2.78%
Treosulfan	288	8	0	8	161.0	2.78%
Olaparib	288	2	5	7	127.5	2.43%
Bleomycin	288	6	0	6	148.0	2.08%
Docetaxel	288	6	0	6	113.0	2.08%
Lapatinib	288	3	3	6	119.5	2.08%
Mitotane	288	5	1	6	176.0	2.08%
Axitinib	288	5	1	6	141.0	2.08%
Abemaciclib	288	5	0	5	168.0	1.74%
Buserelin	288	5	0	5	179.0	1.74%
Sunitinib	288	5	0	5	156.0	1.74%
Lomustine	288	5	0	5	170.0	1.74%
Hydrazine sulfate	288	2	2	4	168.5	1.39%
Goserelin	288	4	0	4	169.0	1.39%
Capecitabine	288	3	1	4	168.0	1.39%
Niraparib	288	1	3	4	120.0	1.39%
Paclitaxel	288	4	0	4	167.0	1.39%

Ifosfamide	288	3	0	3	178.0	1.04%
Capmatinib	288	2	1	3	175.0	1.04%
Encorafenib	288	0	2	2	130.0	0.69%
Anastrozole	288	2	0	2	169.5	0.69%
Hydroxycar-bamide	288	1	1	2	168.0	0.69%
Thiotepa	288	1	1	2	138.0	0.69%
Talazoparib	288	0	2	2	105.0	0.69%
Regorafenib	288	0	1	1	170.0	0.35%
Methotrexate	288	0	1	1	117.0	0.35%
Alectinib	288	0	0	0	173.0	0.00%
Sorafenib	288	0	0	0	177.0	0.00%
DMSO	2304	29	11	40	165.0	1.74%
Untreated wells (no compound added)	1152	15	8	23	162.0	2.00%
Overall	19008	396	127	523	—	2.75%
